# Availability of comparative real-world evidence research in Medicare patients: implications for Centers for Medicare and Medicaid Services drug price negotiations

**DOI:** 10.57264/cer-2023-0125

**Published:** 2023-10-10

**Authors:** Ashley Jaksa, Patrick J Arena, Nicolle Gatto

**Affiliations:** 1 Scientific Research & Strategy, Aetion Inc., Boston, MA 02109, USA; 2 Scientific Research & Strategy, Aetion Inc., New York, NY 10001, USA; 3 Columbia Mailman School of Public Health, New York, NY 10032, USA; 4 Tulane School of Public Health & Tropical Medicine, New Orleans, LA 70112, USA

**Keywords:** comparative effectiveness, comparative outcomes, comparative safety, Medicare, price negotiations, real-world evidence, scoping review

## Abstract

**Aim:**

To evaluate the availability of published comparative real-world evidence (RWE) studies in Medicare patients for the ten drugs set to undergo Centers for Medicare and Medicaid Services (CMS) price negotiations in 2026.

**Materials & methods:**

A scoping review was completed in MEDLINE/PubMed to evaluate the availability of comparative RWE investigations conducted among Medicare-eligible patient populations in the US for the following drugs: apixaban, rivaroxaban, sitagliptin, ibrutinib, empagliflozin, etanercept, dapagliflozin, sacubitril/valsartan, ustekinumab and insulin aspart.

**Results:**

Of the 170 real-world comparative studies identified, 55 (32.4%) used Medicare real-world data (RWD) while 34 (20.0%) used commercial claims data in conjunction with either Medicare Advantage or Medicare Supplementary databases. The number of studies varied considerably by drug with apixaban and rivaroxaban studies accounting for the majority (i.e., 67.1%) of comparative RWE studies. Approximately a third or less of the comparative RWE studies were conducted in CMS RWD per drug.

**Conclusion:**

Our results demonstrate there is a considerable amount of comparative RWE for apixaban, rivaroxaban, and etanercept but limited comparative RWE for the other drugs set to undergo CMS price negotiations in 2026; additionally, our findings set up a number of next steps (e.g., risk of bias assessments) for further exploration of the available evidence base. Overall, CMS and manufacturers should consider proactively generating high-quality comparative RWE studies in the Medicare population to ensure that future price negotiations are based on robust evidence.

## Plain language summary

Following the passage of the Inflation Reduction Act, the Centers for Medicare and Medicaid Services (CMS) will be able to negotiate the price of certain drugs with pharmaceutical companies.

**What is this article about?:**

The US government recently selected the first ten drugs that will be subject to the CMS price negotiations. Here, we investigated the availability of comparative real-world evidence (RWE; evidence from real-world data sources such as electronic health records), which is one component of the clinical evidence that CMS will consider, for the first ten drugs subject to the price negotiations.

**What were the results?:**

Of the 170 comparative RWE studies that we identified in the literature, only about a third (32.4%) were conducted using Medicare data. Additionally, the number of studies varied considerably by drug with apixaban and rivaroxaban studies accounting for the majority (67.1%). We also found that the identified studies examined a wide range of indications, outcomes, and drug comparators.

**What do the results mean?:**

Our findings imply that there is limited comparative RWE on the first ten drugs that will be subject to CMS price negotiations; these results therefore highlight the need for more comparative RWE studies for these drugs in order to ensure that the evidence base for them is as complete as possible.

## Tweetable abstract

A scoping review suggests there is limited comparative #RWE for the first drugs that will be subject to #CMS price negotiations under the #IRA #realworldevidence #Medicare #CER.

Following the passage of the Inflation Reduction Act (IRA) in 2022, the Centers for Medicare and Medicaid Services (CMS) will be able to negotiate Part D drug prices with manufacturers. In August 2023, CMS announced the first ten Part D drugs (represented by eleven brands) selected for negotiations (Table 1) [1]. Additional drugs will be added yearly, including Part B drugs in 2028. Under the IRA, CMS will determine the maximum fair price (MFP), which cannot exceed the lower of the enrollment-weighted price paid by Part D plans or under Part B (net of all price concessions) or a percentage (based on the drug's age) of the average price paid by wholesalers for drugs distributed to non-federal purchasers. The MFP is then adjusted down based on statutory criteria (e.g., comparative clinical effectiveness against therapeutic alternatives) [2]. As part of the negotiation process, CMS *“intends to review existing literature and real-world evidence”* (RWE) to explore the unmet need, identify therapeutic alternatives, and compare a given drug to those alternatives in order to evaluate its place in the broader clinical landscape. Moreover, CMS has indicated that it will focus on rigorous studies in the Medicare population [3]. RWE studies (i.e., evidence generated from real-world data [RWD] like claims data, electronic health records, registries, etc. [4]) are one of many possible sources of evidence available to support price negotiations. However, because of the timing of the price negotiations (i.e., 7–11 years post-regulatory approval [3]), the decreasing likelihood of additional randomized controlled trials (RCTs) post-launch [5], and the fact that older patients tend to be excluded from RCTs [6], RWE studies are expected to be a critical component of CMS's clinical evaluation a drug relative to its therapeutic alternatives.

**Table 1.** Ten drugs selected for CMS price negotiations in 2026.

Drug brand name	Drug generic name	FDA approval^†^
Eliquis	Apixaban	2012
Jardiance	Empagliflozin	2014
Xarelto	Rivaroxaban	2011
Januvia	Sitagliptin	2006
Farxiga	Dapagliflozin	2014
Entresto	Sacubitril/valsartan	2015
Enbrel	Etanercept	1998
Imbruvica	Ibrutinib	2013
Stelera	Ustekinumab	2009
Fiasp; Fiasp FlexTouch; Fiasp PenFill; NovoLog; NovoLog FlexPen; NovoLog PenFill	Insulin aspart	NovoLog, 2000 Fiasp, 2017

† All FDA approval dates retrieved from Drugs@FDA database: https://www.accessdata.fda.gov/scripts/cder/daf/index.cfm.

Insulin aspart is represented by two different brands (i.e., Fiasp and NovoLog), which are bundled together by CMS and here.

CMS: Centers for Medicare and Medicaid Services; FDA: Food and Drug Administration.

Clinical evidence gaps are often cited in drug price negotiations by health technology assessment bodies and other payers as key contributors to the uncertainty surrounding the value of a product and thus make price negotiations challenging [7]. Hence, a natural question in the context of the IRA is whether relevant evidence (including comparative RWE studies) that addresses important evidence gaps will be available in the literature at the time of the CMS price negotiations. Relatedly, key stakeholders are unclear whether CMS and/or manufacturers will need to conduct appropriate analyses in order to strengthen the evidence base if applicable studies have not already been produced. We therefore conducted a scoping review to evaluate the availability of published comparative RWE studies in Medicare patients for the ten drugs set to undergo CMS price negotiations in 2026 (Table 1).

## Methods

### Population, intervention, comparator & outcome (PICO)

As this scoping review focused on the context of the IRA [8], we limited our framework to only include RWE investigations conducted among Medicare-eligible patient populations in the US. We therefore defined our population of interest as Medicare-aged patients (i.e., patients ≥65 years of age) as well as other Medicare-eligible individuals (i.e., those with qualifying disabilities and/or end-stage renal disease) in the US; in order to ensure that RWE investigations were included, we only allowed for studies among these patients that used some form of RWD, such as claims data, electronic health records, and/or registry data. If any one of the ten CMS-selected drugs was used as either the intervention or control and was compared with any other drug, the study was included; in situations where both the intervention and the control were any of the CMS-selected drugs of interest (e.g., apixaban vs rivaroxaban), the study was also included. In order to select for robust comparative investigations, we only included analyses that employed appropriate epidemiologic and statistical methodology (e.g., propensity score matching, inverse probability weighting, multivariable regression, etc.) to control confounding when comparing at least one drug against at least one other drug. Last, there were no explicit pre-specified outcomes of interest; rather, we were interested in studies that assessed comparative effectiveness, comparative safety, comparative adherence, comparative costs and/or any other relevant outcome from a comparative perspective.

### Search strategy

We searched Medical Literature Analysis and Retrieval System Online (MEDLINE)/PubMed for articles published any time up to 29 August 2023 (inclusive) and tailored our combination of subject headings/controlled vocabulary (e.g., Medical Subject Headings) and keyword fields (e.g., publication type) to identify potentially relevant publications. The overall primary search strategy was formulated according to the PICO model [9]; the search string combination used along with its corresponding PICO components are provided in Supplementary Table 1. Additionally, due to the limited RWE literature on insulin aspart specifically, we conducted a secondary search that investigated insulin broadly. The secondary search strategy used the same approach as our primary search strategy but replaced the ten CMS-selected drugs with ‘insulin’ only. However, due to the large number of studies yielded by this approach, we only included a ∼10% random sample of the results for data screening and extraction. Google Sheets was used to save/organize all references and provide an interface for screening.

### Data screening & extraction

Two authors (AJ and PJA) performed abstract screening to select for comparative RWE studies evaluating one of the pre-specified drugs of interest among Medicare-eligible patients in the US for both the primary and secondary search strategies. To ensure consistency in the review process, AJ and PJA each reviewed a random selection of fifty abstracts to confirm they were using the same criteria in their assessments; any discrepancies were resolved through consensus discussion between these two reviewers in order to align on the assessment process. From there, AJ reviewed one-half of the remaining articles while PJA reviewed the other half (in order to allow for a more rapid turnaround due to the time-sensitive nature of the research question). The following data elements were extracted via a standardized form: first author, journal name, publication year, drug(s) assessed (i.e., the pre-specified drugs of interest), comparative nature (i.e., yes or no), Medicare-eligible population (i.e., yes or no), data type (i.e., CMS RWD, commercial RWD with Medicare Advantage or Medicare Supplementary databases, or other RWD), data source (if other RWD), indication(s)/therapeutic area(s), comparator(s) and outcome(s). Due to the nature of our research question, identified studies were not assessed for their quality/validity (beyond ensuring confounding control methods were applied); likewise, publication bias was also not assessed.

## Results

**Figure 1.**
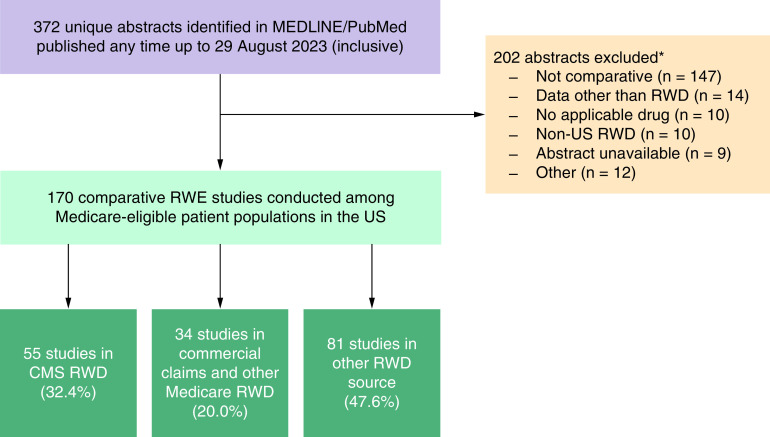
Study selection flow chart for primary search. *It was possible for abstracts to have more than one reason for exclusion; however, only the primary reason for exclusion for each abstract is used here. CMS: Centers for Medicare and Medicaid Services; MEDLINE: Medical Literature Analysis and Retrieval System Online; RWD: Real-world data; RWE: Real-world evidence.

We initially identified 372 unique RWE studies that appeared to include a drug of interest and to employ confounding control methods for drug-to-drug comparisons according to the primary search strategy; however, 202 records were excluded after title/abstract review (54.3%; Figure 1). Most records were excluded because they were not comparative per our definition (n = 147); other reasons for exclusion included using data other than RWD, such as RCT data (n = 14), no examination of an applicable drug (n = 10), utilization of non-US RWD (n = 10), or unavailable abstract (n = 9). Of the 170 applicable studies, 81 studies (47.6%) were conducted in a non-Medicare RWD source, such as an institution's internal medical records management system, a commercial RWD source (e.g., Optum Clinformatics Data Mart), or RWD from the US Department of Veterans Affairs, 55 (32.4%) used Medicare RWD, and 34 (20.0%) used commercial claims data in conjunction with either Medicare Advantage or Medicare Supplementary databases (Figure 1). Please refer to Supplementary Table 2 for a complete list of the 170 identified studies.

The number of comparative studies varied considerably by drug (Table 2). More specifically, there were several comparative studies of apixaban and/or rivaroxaban (both included: n = 61; rivaroxaban only: n = 34; apixaban only: n = 19) and etanercept (n = 26). Apixaban and/or rivaroxaban studies accounted for approximately a third of all the comparative studies identified (67.1%; Table 3). The three diabetes drugs (i.e., empagliflozin, dapagliflozin, and sitagliptin) had a total of 17 comparative studies, but few studies of ibrutinib (n = 6), sacubitril/valsartan (n = 5), and ustekinumab (n = 2) were identified; furthermore, no relevant studies were found for insulin aspart according to the primary search strategy (Table 2). Approximately a third of the comparative studies per drug were conducted in CMS RWD. Apixaban and/or rivaroxaban studies also accounted for the majority of comparative studies conducted in CMS RWD.

**Table 2.** Scoping review results, by drug and RWD type, according to the primary search strategy.

Drug(s)	CMS RWD	Commercial with other Medicare RWD	Other RWD	Overall^†^
Apixaban and/or rivaroxaban	40 (35.1%)	21 (18.4%)	53 (46.5%)	114 (100%)
*Both apixaban and rivaroxaban	27 (67.5%)	6 (28.6%)	28 (52.8%)	61 (53.5%)
*Apixaban only	8 (20.0%)	3 (14.3%)	8 (15.1%)	19 (16.7%)
*Rivaroxaban only	5 (12.5%)	12 (57.1%)	17 (32.1%)	34 (29.8%)
Empagliflozin, dapagliflozin, and/or sitagliptin	6 (35.3%)	6 (35.3%)	5 (29.4%)	17 (100%)
*Both dapagliflozin and sitagliptin	1 (16.7%)	0 (0.0%)	0 (0.0%)	1 (5.9%)
*Both empagliflozin and dapagliflozin	0 (0.0%)	0 (0.0%)	1 (20.0%)	1 (5.9%)
*Both empagliflozin and sitagliptin	2 (33.3%)	0 (0.0%)	0 (0.0%)	2 (11.8%)
*Dapagliflozin only	2 (33.3%)	0 (0.0%)	2 (40.0%)	4 (23.5%)
*Empagliflozin only	0 (0.0%)	1 (16.7%)	0 (0.0%)	1 (5.9%)
*Sitagliptin only	1 (16.7%)	5 (83.3%)	2 (40.0%)	8 (47.1%)
Etanercept	8 (30.8%)	6 (23.1%)	12 (46.2%)	26 (100%)
Ibrutinib	0 (0.0%)	1 (16.7%)	5 (83.3%)	6 (100%)
Insulin aspart	–	–	–	–
Sacubitril/valsartan	1 (20.0%)	0 (0.0%)	4 (80.0%)	5 (100%)
Ustekinumab	0 (0.0%)	0 (0.0%)	2 (100%)	2 (100%)

† Sum of ‘CMS RWD’, ‘Commercial with Other Medicare RWD’ and ‘Other RWD’ columns.

*Parentheticals of cells in this row represent column percentages.

No studies were identified for insulin aspart so associated information is not provided here.

CMS: Centers for Medicare and Medicaid Services; RWD: Real-world data.

**Table 3.** Number of identified studies and summary of primary indication/comparator/outcome information, by drug, according to the primary search strategy.

Drug(s)	Studies, n (% of total)	Primary indication(s)	Primary comparator(s)	Primary outcome(s)
Apixaban and/or rivaroxaban	114 (67.1%)	Atrial fibrillation, VTE, DVT, and pulmonary embolism	Dabigatran and warfarin	Stroke, bleeding, mortality, recurrent VTE, HCRU, costs and adherence
Empagliflozin, dapagliflozin, and/or sitagliptin	17 (10.0%)	Diabetes	Canagliflozin and other glucose-lowering drugs	General safety, costs and HCRU
Etanercept	26 (15.3%)	RA, psoriasis, IBD, psoriatic arthritis, and ankylosing spondylitis	Non-biologic agents and other biologic agents	General safety, HCRU and adherence
Ibrutinib	6 (3.5%)	Chronic lymphocytic leukemia	Chemoimmunotherapy	Survival, costs and HCRU
Insulin aspart	0 (0%)	–	–	–
Sacubitril/valsartan	5 (2.9%)	Heart failure	ACE inhibitors and angiotensin receptor blockers	Survival and costs
Ustekinumab	2 (1.2%)	Psoriasis and IBD	Other monoclonal antibody medications	Survival and infection-related hospitalization

No studies were identified for insulin aspart so associated primary indication/comparator/outcome is not provided here.

ACE: Angiotensin-converting enzyme; DVT: Deep vein thrombosis; HCRU: Healthcare resource utilization; IBD: Inflammatory bowel disease; RA: Rheumatoid arthritis; VTE: Venous thromboembolism.

The identified studies examined a wide spectrum of indications, outcomes, and comparators (Table 3). For instance, apixaban, rivaroxaban and etanercept studies spanned multiple patient populations reflective of their multiple indications approved by the US FDA. For apixaban and rivaroxaban, the majority of comparative studies in CMS RWD were in patients with atrial fibrillation (Supplementary Table 3). Primary outcomes ranged from clinical effectiveness (e.g., survival and stroke) and general safety end points to costs, adherence and healthcare resource utilization (HCRU). Furthermore, most drugs were compared with several other therapies (e.g., dabigatran and warfarin for apixaban and/or rivaroxaban as well as canagliflozin and other glucose-lower drugs for empagliflozin, dapagliflozin, and/or sitagliptin) across the comparative studies identified. Drugs on CMS's list were also often compared with each other: apixaban was compared with rivaroxaban in 61 studies, while empagliflozin was compared with sitagliptin in two studies and compared with dapagliflozin in one study (Table 2). Other comparators were often in a similar drug class to the drug of interest (e.g., apixaban and rivaroxaban were often compared with another direct oral anticoagulant, dabigatran). For the apixaban and rivaroxaban comparative studies in CMS RWD, apixaban was compared with rivaroxaban in 27 studies, to warfarin in 33 studies, to dabigatran in 22 studies and to edoxaban in one study; similarly, rivaroxaban was compared with warfarin in 30 studies, to dabigatran in 25 studies and to edoxaban in one study (Supplementary Table 4).

Lastly, in the secondary search, we identified 637 unique studies that appeared to investigate insulin broadly but only reviewed the title/abstract for the 60 studies selected from the random sampling procedure. Among these randomly selected studies, we determined that only four were RWE studies that included insulin and employed confounding control methods for drug-to-drug comparisons among elderly patients in the US. None of these four studies were conducted in CMS RWD: one used commercial claims data in conjunction with either Medicare Advantage or Medicare Supplementary databases, while the remaining three were in other RWD sources. The primary indication and outcome for all four studies was diabetes and glycemic control, respectively; two of the studies compared insulin glargine versus insulin detemir, while the remaining two studies evaluated different methods of insulin administration (e.g., wearable insulin delivery compared with multiple daily injections; data not shown).

## Discussion

As part of the drug pricing negotiation process, CMS will be evaluating therapeutic advances as well as the comparative effectiveness of a given drug compared with its therapeutic alternative(s) in the Medicare patient population and the extent to which the drug addresses unmet needs. These evaluations will largely rely on published comparative studies, of which RWE investigations will likely play a critical role. In this investigation, we examined the published literature to determine the scope of comparative RWE that may be applicable in the upcoming price negotiations. While our findings demonstrated that there is considerable comparative RWE for apixaban, rivaroxaban, and etanercept, the remaining drugs set to undergo price negotiations had more limited comparative RWE. Indeed, when focusing specifically on the CMS patient population, we found that the evidence base was reduced by more than half.

Additional factors, including the appropriate therapeutic alternative and the robustness of the studies, might further limit the evidence base applicable for price negotiations. For example, a number of the apixaban/rivaroxaban studies used warfarin as the primary comparator, a situation that could appreciably reduce the evidence base if warfarin is not deemed a relevant therapeutic alternative by CMS. Similarly, the robustness of the evidence will almost certainly play an important role. While our focus was only on the availability of RWE studies, the evidence base will likely be further diminished since CMS will rely on robust and valid evidence [10], and RWE investigations can produce biased results when appropriate methods are not used. For instance, a previous study found that 81% of seventy-five published comparative RWE studies had at least one major methodological flaw that potentially undermined their validity [11]. Moreover, this limited availability of relevant comparative RWE studies could indicate a similar trend among other types of comparative studies (e.g., RCTs), especially since RCT studies are less likely to be conducted post-launch [5]. Thus, even though RCTs may be of a higher evidence grade [12], it may still be the case that the overall comparative evidence base for these drugs is limited, thereby leading to uncertainty over how such a situation will impact the CMS price negotiations.

To answer the research question, “*What is the availability of published comparative RWE studies in Medicare patients?”*, we performed a scoping review as opposed to a systematic review. Consequently, our findings highlight a number of next steps that can further explore the available evidence base and the utility of this evidence base for CMS price negotiations. First, assessing the validity and quality of the identified studies will be a critical aspect in determining how useful each study will actually be for CMS negotiations. Future studies in this area should thus employ risk of bias tools, such as the “Risk Of Bias In Non-randomised Studies – of Interventions” [13], to characterize the validity and quality of these potentially relevant RWE studies. Likewise, subsequent investigations should further evaluate differences in study populations, indications of interest, design elements, outcomes (including the specific end points), and comparators in each study; more detailed (qualitative and quantitative) information on these variables would thus allow stakeholders to better understand how many studies CMS may ultimately find applicable according to their specifications, especially in the context of drugs like apixaban/rivaroxaban that had a large number of studies. Such information could also help better highlight the versatility of RWE to address a variety of comparative outcomes (as was observed here, albeit at a much broader level). Lastly, we also recommend that additional databases (e.g., Embase) be used in order to ensure that a more comprehensive approach is achieved.

## Conclusion

Overall, our findings highlight the need for more comparative RWE studies for drugs set to undergo CMS price negotiations in order to ensure that the evidence base for these drugs is as complete as possible. Though our primary analysis considered only the first ten drugs set to undergo price negotiations in 2026, we believe it is reasonable to assume our findings can be generalized to the additional drugs that will undergo price negotiations in subsequent years. Thus, manufacturers, CMS, and other researchers should consider proactively generating comparative RWE studies in Medicare patients to address relevant evidence gaps. Manufacturers have a limited window to submit evidence to CMS once their product has been identified for price negotiations and the manufacturer agrees to participate (i.e., ~30 days); advanced preparation of comparative studies is therefore essential.

## Summary points

- The Centers for Medicare and Medicaid Services (CMS) has stated that real-world evidence (RWE) will be considered in drug price negotiations; additionally, CMS has said that it will consider comparative RWE studies in the Medicare patient population. Thus, RWE will likely be an important piece of the evidence base.
- In an evaluation of the ten drugs set to undergo CMS price negotiations in 2026, we found that the comparative RWE base in Medicare patients was limited. Only a few drugs (e.g., apixaban, rivaroxaban, and etanercept) had more than two comparative studies performed in CMS real-world data.
- In order to ensure a robust evidence base is available for CMS price negotiations, CMS and manufacturers should consider proactively generating high-quality comparative RWE studies in the Medicare patient population.
