# Supplementary material for: Availability of comparative real-world evidence research in Medicare patients: implications for Centers for Medicare and Medicaid Services drug price negotiations
Source: J Comp Eff Res. 2023 Oct 10;12(11):e230125. doi: 10.57264/cer-2023-0125 (PMC10690442; doi:10.57264/cer-2023-0125)
Supplement: Supplementary file 1 [file cer-12-230125-s1.docx]

**Supplemental Table 1. Primary search strategy for MEDLINE/PubMed**

| **PICO Category** | **Associated Concept** | **Search Terms** |
| --- | --- | --- |
| Population | RWE studies among Medicare-aged patients (i.e., patients ≥ 65 years of age) as well as other Medicare-eligible individuals (i.e., those with qualifying disabilities and/or end-stage renal disease) in the US | ("Aged"[Mesh] OR "Aged, 80 and over"[Mesh] OR "Medicare"[Mesh]) AND "United States" AND ("Comparative Effectiveness Research"[Mesh] OR "Retrospective Studies"[Mesh] OR "Observational Studies as Topic"[Mesh] OR "real-world" OR "Observational Study"[Publication Type]) |
| Intervention | Apixaban, rivaroxaban, sitagliptin, ibrutinib, empagliflozin, etanercept, dapagliflozin, sacubitril/valsartan, ustekinumab, and/or insulin aspart | ("Apixaban" OR "Rivaroxaban" OR "Sitagliptin" OR "Ibrutinib" OR "Empagliflozin" OR "Etanercept" OR "Dapagliflozin" OR "Sacubitril/valsartan" OR "Ustekinumab" OR "Insulin aspart") |
| Comparator | Any drug (including any of the interventions above) | *Not applicable – however, all identified abstracts were screened to ensure that the associated studies were comparative against at least one other drug and that the comparison used appropriate epidemiologic/statistical methodology to control confounding.* |
| Outcome(s) | Comparative effectiveness, comparative safety, comparative adherence, comparative costs, and/or any other relevant outcome from a comparative perspective | *Not applicable – however, all identified abstracts were screened to ensure that the associated studies were comparative for a given outcome against at least one other drug.* |
| MEDLINE = Medical Literature Analysis and Retrieval System Online, PICO = population, intervention, comparator, and outcome,  RWE = real-world evidence, US = United States. | | |

**Supplemental Table 2. List of identified studies from the primary search**

| **Drug(s)** | **PMID** | **First Author** | **Title** | **Citation** |
| --- | --- | --- | --- | --- |
| Apixaban & rivaroxaban | 28854073 | Adeboyeje G | Major Bleeding Risk During Anticoagulation with Warfarin, Dabigatran, Apixaban, or Rivaroxaban in Patients with Nonvalvular Atrial Fibrillation | J Manag Care Spec Pharm. 2017 Sep;23(9):968-978. doi: 10.18553/jmcp.2017.23.9.968. |
| Apixaban & rivaroxaban | 32291717 | Alcusky M | Comparative Safety and Effectiveness of Direct-Acting Oral Anticoagulants Versus Warfarin: a National Cohort Study of Nursing Home Residents | J Gen Intern Med. 2020 Aug;35(8):2329-2337. doi: 10.1007/s11606-020-05777-3. Epub 2020 Apr 6. |
| Apixaban & rivaroxaban | 29047304 | Amin A | Real-world comparison of all-cause hospitalizations, hospitalizations due to stroke and major bleeding, and costs for non-valvular atrial fibrillation patients prescribed oral anticoagulants in a US health plan | J Med Econ. 2018 Mar;21(3):244-253. doi: 10.1080/13696998.2017.1394866. Epub 2017 Nov 20. |
| Apixaban & rivaroxaban | 30156450 | Amin A | A Real-World Observational Study of Hospitalization and Health Care Costs Among Nonvalvular Atrial Fibrillation Patients Prescribed Oral Anticoagulants in the U.S. Medicare Population | J Manag Care Spec Pharm. 2018 Sep;24(9):911-920. doi: 10.18553/jmcp.2018.24.9.911. |
| Apixaban & rivaroxaban | 30908512 | Amin A | Effectiveness and safety of oral anticoagulants in older adults with non-valvular atrial fibrillation and heart failure | PLoS One. 2019 Mar 25;14(3):e0213614. doi: 10.1371/journal.pone.0213614. eCollection 2019. |
| Apixaban & rivaroxaban | 30924051 | Amin A | Comparative clinical outcomes between direct oral anticoagulants and warfarin among elderly patients with non-valvular atrial fibrillation in the CMS medicare population | J Thromb Thrombolysis. 2019 Aug;48(2):240-249. doi: 10.1007/s11239-019-01838-5. |
| Apixaban & rivaroxaban | 32347184 | Amin A | A Real-World Observational Study of Hospitalization and Health Care Costs Among Nonvalvular Atrial Fibrillation Patients Prescribed Oral Anticoagulants in the U.S. Medicare Population | J Manag Care Spec Pharm. 2020 May;26(5):639-651. doi: 10.18553/jmcp.2020.26.5.639. |
| Apixaban & rivaroxaban | 35993487 | Amin A | Risk of stroke/systemic embolism, major bleeding, and associated costs in non-valvular atrial fibrillation patients who initiated apixaban, dabigatran, or rivaroxaban compared with warfarin in the United States medicare population: updated analysis | Curr Med Res Opin. 2022 Dec;38(12):2131-2140. doi: 10.1080/03007995.2022.2115772. Epub 2022 Aug 30. |
| Apixaban & rivaroxaban | 30499067 | Baker CL | Comparison of Drug Switching and Discontinuation Rates in Patients with Nonvalvular Atrial Fibrillation Treated with Direct Oral Anticoagulants in the United States | Adv Ther. 2019 Jan;36(1):162-174. doi: 10.1007/s12325-018-0840-8. Epub 2018 Nov 29. |
| Apixaban & rivaroxaban | 33404923 | Briasoulis A | Comparative Effectiveness and Safety of Direct Oral Anticoagulants in Obese Patients with Atrial Fibrillation | Cardiovasc Drugs Ther. 2021 Apr;35(2):261-272. doi: 10.1007/s10557-020-07126-2. Epub 2021 Jan 6. |
| Apixaban & rivaroxaban | 27783556 | Brown JD | Adherence to Rivaroxaban, Dabigatran, and Apixaban for Stroke Prevention in Incident, Treatment-Naïve Nonvalvular Atrial Fibrillation | J Manag Care Spec Pharm. 2016 Nov;22(11):1319-1329. doi: 10.18553/jmcp.2016.22.11.1319. |
| Apixaban & rivaroxaban | 28854077 | Brown JD | Adherence to Rivaroxaban, Dabigatran, and Apixaban for Stroke Prevention for Newly Diagnosed and Treatment-Naive Atrial Fibrillation Patients: An Update Using 2013-2014 Data | J Manag Care Spec Pharm. 2017 Sep;23(9):958-967. doi: 10.18553/jmcp.2017.23.9.958. |
| Apixaban & rivaroxaban | 31549883 | Chowdhury R | Healthcare resource utilization and expenditures among newly-diagnosed elderly non-valvular atrial fibrillation patients initiating oral anticoagulants | J Med Econ. 2019 Dec;22(12):1338-1350. doi: 10.1080/13696998.2019.1672698. Epub 2019 Oct 12. |
| Apixaban & rivaroxaban | 27633045 | Coleman CI | Real-world evidence of stroke prevention in patients with nonvalvular atrial fibrillation in the United States: the REVISIT-US study | Curr Med Res Opin. 2016 Dec;32(12):2047-2053. doi: 10.1080/03007995.2016.1237937. Epub 2016 Sep 20. |
| Apixaban & rivaroxaban | 34871048 | Dawwas GK | Risk for Recurrent Venous Thromboembolism and Bleeding With Apixaban Compared With Rivaroxaban: An Analysis of Real-World Data | Ann Intern Med. 2022 Jan;175(1):20-28. doi: 10.7326/M21-0717. Epub 2021 Dec 7. |
| Apixaban & rivaroxaban | 31112292 | Deitelzweig S | Comparisons between Oral Anticoagulants among Older Nonvalvular Atrial Fibrillation Patients | J Am Geriatr Soc. 2019 Aug;67(8):1662-1671. doi: 10.1111/jgs.15956. Epub 2019 May 21. |
| Apixaban & rivaroxaban | 36456387 | Deitelzweig S | Delaying clinical events among patients with non-valvular atrial fibrillation treated with oral anticoagulants: Insights from the ARISTOPHANES study | Eur J Intern Med. 2023 Feb;108:37-42. doi: 10.1016/j.ejim.2022.10.021. Epub 2022 Nov 28. |
| Apixaban & rivaroxaban | 36527598 | Dhamane AD | Effectiveness and Safety of Direct Oral Anticoagulants Among Patients with Non-valvular Atrial Fibrillation and Multimorbidity | Adv Ther. 2023 Mar;40(3):887-902. doi: 10.1007/s12325-022-02387-9. Epub 2022 Dec 17. |
| Apixaban & rivaroxaban | 29166800 | Gilligan AM | Comparison of all-cause costs and healthcare resource use among patients with newly-diagnosed non-valvular atrial fibrillation newly treated with oral anticoagulants | Curr Med Res Opin. 2018 Feb;34(2):285-295. doi: 10.1080/03007995.2017.1409425. Epub 2017 Nov 30. |
| Apixaban & rivaroxaban | 30639551 | Graham DJ | Comparative Stroke, Bleeding, and Mortality Risks in Older Medicare Patients Treated with Oral Anticoagulants for Nonvalvular Atrial Fibrillation | Am J Med. 2019 May;132(5):596-604.e11. doi: 10.1016/j.amjmed.2018.12.023. Epub 2019 Jan 9. |
| Apixaban & rivaroxaban | 30212268 | Gupta K | Real-World Comparative Effectiveness, Safety, and Health Care Costs of Oral Anticoagulants in Nonvalvular Atrial Fibrillation Patients in the U.S. Department of Defense Population | J Manag Care Spec Pharm. 2018 Nov;24(11):1116-1127. doi: 10.18553/jmcp.2018.17488. Epub 2018 Sep 13. |
| Apixaban & rivaroxaban | 31195999 | Gupta K | Effectiveness and safety of direct oral anticoagulants compared to warfarin in treatment naïve non-valvular atrial fibrillation patients in the US Department of defense population | BMC Cardiovasc Disord. 2019 Jun 13;19(1):142. doi: 10.1186/s12872-019-1116-1. |
| Apixaban & rivaroxaban | 29681367 | Hernandez I | Effectiveness and Safety of Direct Oral Anticoagulants and Warfarin, Stratified by Stroke Risk in Patients With Atrial Fibrillation | Am J Cardiol. 2018 Jul 1;122(1):69-75. doi: 10.1016/j.amjcard.2018.03.012. Epub 2018 Mar 28. |
| Apixaban & rivaroxaban | 30449001 | Howe Z | Bleeding rates of Veterans taking apixaban or rivaroxaban for atrial fibrillation or venous thromboembolism | J Thromb Thrombolysis. 2019 Feb;47(2):280-286. doi: 10.1007/s11239-018-1770-7. |
| Apixaban & rivaroxaban | 34198049 | Jin MC | Hemorrhage risk of direct oral anticoagulants in real-world venous thromboembolism patients | Thromb Res. 2021 Aug;204:126-133. doi: 10.1016/j.thromres.2021.06.015. Epub 2021 Jun 27. |
| Apixaban & rivaroxaban | 29042362 | Jun M | Comparative safety of direct oral anticoagulants and warfarin in venous thromboembolism: multicentre, population based, observational study | BMJ. 2017 Oct 17;359:j4323. doi: 10.1136/bmj.j4323. |
| Apixaban & rivaroxaban | 35613184 | Khan AM | Survival outcomes with warfarin compared with direct oral anticoagulants in cancer-associated venous thromboembolism in the United States: A population-based cohort study | PLoS Med. 2022 May 25;19(5):e1004012. doi: 10.1371/journal.pmed.1004012. eCollection 2022 May. |
| Apixaban & rivaroxaban | 34280330 | Kim DH | Frailty and Clinical Outcomes of Direct Oral Anticoagulants Versus Warfarin in Older Adults With Atrial Fibrillation : A Cohort Study | Ann Intern Med. 2021 Sep;174(9):1214-1223. doi: 10.7326/M20-7141. Epub 2021 Jul 20. |
| Apixaban & rivaroxaban | 36976562 | Lin KJ | Comparative Effectiveness and Safety of Oral Anticoagulants by Dementia Status in Older Patients With Atrial Fibrillation | JAMA Netw Open. 2023 Mar 1;6(3):e234086. doi: 10.1001/jamanetworkopen.2023.4086. |
| Apixaban & rivaroxaban | 27550177 | Lip GY | Major bleeding risk among non-valvular atrial fibrillation patients initiated on apixaban, dabigatran, rivaroxaban or warfarin: a "real-world" observational study in the United States | Int J Clin Pract. 2016 Sep;70(9):752-63. doi: 10.1111/ijcp.12863. Epub 2016 Aug 23. |
| Apixaban & rivaroxaban | 29709012 | Lip GYH | Discontinuation risk comparison among 'real-world' newly anticoagulated atrial fibrillation patients: Apixaban, warfarin, dabigatran, or rivaroxaban | PLoS One. 2018 Apr 30;13(4):e0195950. doi: 10.1371/journal.pone.0195950. eCollection 2018. |
| Apixaban & rivaroxaban | 32602228 | Lip GYH | Oral anticoagulants for nonvalvular atrial fibrillation in frail elderly patients: insights from the ARISTOPHANES study | J Intern Med. 2021 Jan;289(1):42-52. doi: 10.1111/joim.13140. Epub 2020 Jul 16. |
| Apixaban & rivaroxaban | 33010157 | Lip GYH | Effectiveness and safety of oral anticoagulants among non-valvular atrial fibrillation patients with polypharmacy | Eur Heart J Cardiovasc Pharmacother. 2021 Sep 21;7(5):405-414. doi: 10.1093/ehjcvp/pvaa117. |
| Apixaban & rivaroxaban | 34398204 | Lip GYH | Oral Anticoagulants for Nonvalvular Atrial Fibrillation in Patients With High Risk of Gastrointestinal Bleeding | JAMA Netw Open. 2021 Aug 2;4(8):e2120064. doi: 10.1001/jamanetworkopen.2021.20064. |
| Apixaban & rivaroxaban | 35579733 | Lip GYH | Effectiveness and safety of oral anticoagulants in non-valvular atrial fibrillation patients with prior bleeding events: a retrospective analysis of administrative claims databases | J Thromb Thrombolysis. 2022 Jul;54(1):33-46. doi: 10.1007/s11239-022-02660-2. Epub 2022 May 17. |
| Apixaban & rivaroxaban | 29807001 | Lopes RD | Effectiveness and Safety of Anticoagulants in Adults with Non-valvular Atrial Fibrillation and Concomitant Coronary/Peripheral Artery Disease | Am J Med. 2018 Sep;131(9):1075-1085.e4. doi: 10.1016/j.amjmed.2018.05.007. Epub 2018 May 26. |
| Apixaban & rivaroxaban | 33667438 | Lopes RD | Clinical and Economic Outcomes Among Nonvalvular Atrial Fibrillation Patients With Coronary Artery Disease and/or Peripheral Artery Disease | Am J Cardiol. 2021 Jun 1;148:69-77. doi: 10.1016/j.amjcard.2021.02.021. Epub 2021 Mar 3. |
| Apixaban & rivaroxaban | 30919942 | Lutsey PL | Risk of hospitalised bleeding in comparisons of oral anticoagulant options for the primary treatment of venous thromboembolism | Br J Haematol. 2019 Jun;185(5):903-911. doi: 10.1111/bjh.15857. Epub 2019 Mar 28. |
| Apixaban & rivaroxaban | 31764956 | Lutsey PL | Association of Anticoagulant Therapy With Risk of Fracture Among Patients With Atrial Fibrillation | JAMA Intern Med. 2020 Feb 1;180(2):245-253. doi: 10.1001/jamainternmed.2019.5679. |
| Apixaban & rivaroxaban | 28730619 | Manzoor BS | Real-World Adherence and Persistence with Direct Oral Anticoagulants in Adults with Atrial Fibrillation | Pharmacotherapy. 2017 Oct;37(10):1221-1230. doi: 10.1002/phar.1989. Epub 2017 Sep 6. |
| Apixaban & rivaroxaban | 29654196 | Martinez BK | Effectiveness and Safety of Apixaban, Dabigatran, and Rivaroxaban Versus Warfarin in Frail Patients With Nonvalvular Atrial Fibrillation | J Am Heart Assoc. 2018 Apr 13;7(8):e008643. doi: 10.1161/JAHA.118.008643. |
| Apixaban & rivaroxaban | 29621248 | McHorney CA | Adherence to rivaroxaban versus apixaban among patients with non-valvular atrial fibrillation: Analysis of overall population and subgroups of prior oral anticoagulant users | PLoS One. 2018 Apr 5;13(4):e0194099. doi: 10.1371/journal.pone.0194099. eCollection 2018. |
| Apixaban & rivaroxaban | 32517580 | Mentias A | Comparative Effectiveness of Rivaroxaban, Apixaban, and Warfarin in Atrial Fibrillation Patients With Polypharmacy | Stroke. 2020 Jul;51(7):2076-2086. doi: 10.1161/STROKEAHA.120.029541. Epub 2020 Jun 10. |
| Apixaban & rivaroxaban | 32897766 | Milentijevic D | Healthcare costs of NVAF patients treated with rivaroxaban and apixaban in the US | J Med Econ. 2020 Nov;23(11):1365-1374. doi: 10.1080/13696998.2020.1821038. Epub 2020 Sep 29. |
| Apixaban & rivaroxaban | 35176074 | Munir MB | Contemporary clinical and economic outcomes among oral anticoagulant treated and untreated elderly patients with atrial fibrillation: Insights from the United States Medicare database | PLoS One. 2022 Feb 17;17(2):e0263903. doi: 10.1371/journal.pone.0263903. eCollection 2022. |
| Apixaban & rivaroxaban | 27938741 | Noseworthy PA | Direct Comparison of Dabigatran, Rivaroxaban, and Apixaban for Effectiveness and Safety in Nonvalvular Atrial Fibrillation | Chest. 2016 Dec;150(6):1302-1312. doi: 10.1016/j.chest.2016.07.013. Epub 2016 Sep 28. |
| Apixaban & rivaroxaban | 32329770 | Pham P | Association of Oral Anticoagulants and Verapamil or Diltiazem With Adverse Bleeding Events in Patients With Nonvalvular Atrial Fibrillation and Normal Kidney Function | JAMA Netw Open. 2020 Apr 1;3(4):e203593. doi: 10.1001/jamanetworkopen.2020.3593. |
| Apixaban & rivaroxaban | 30512099 | Ray WA | Association of Oral Anticoagulants and Proton Pump Inhibitor Cotherapy With Hospitalization for Upper Gastrointestinal Tract Bleeding | JAMA. 2018 Dec 4;320(21):2221-2230. doi: 10.1001/jama.2018.17242. |
| Apixaban & rivaroxaban | 34932078 | Ray WA | Association of Rivaroxaban vs Apixaban With Major Ischemic or Hemorrhagic Events in Patients With Atrial Fibrillation | JAMA. 2021 Dec 21;326(23):2395-2404. doi: 10.1001/jama.2021.21222. |
| Apixaban & rivaroxaban | 30103250 | Roetker NS | All-Cause Mortality Risk with Direct Oral Anticoagulants and Warfarin in the Primary Treatment of Venous Thromboembolism | Thromb Haemost. 2018 Sep;118(9):1637-1645. doi: 10.1055/s-0038-1668521. Epub 2018 Aug 13. |
| Apixaban & rivaroxaban | 29378726 | Shah S | Comparative effectiveness of direct oral anticoagulants and warfarin in patients with cancer and atrial fibrillation | Blood Adv. 2018 Feb 13;2(3):200-209. doi: 10.1182/bloodadvances.2017010694. |
| Apixaban & rivaroxaban | 29954737 | Siontis KC | Outcomes Associated With Apixaban Use in Patients With End-Stage Kidney Disease and Atrial Fibrillation in the United States | Circulation. 2018 Oct 9;138(15):1519-1529. doi: 10.1161/CIRCULATIONAHA.118.035418. |
| Apixaban & rivaroxaban | 27978942 | Steinberg BA | Off-Label Dosing of Non-Vitamin K Antagonist Oral Anticoagulants and Adverse Outcomes: The ORBIT-AF II Registry | J Am Coll Cardiol. 2016 Dec 20;68(24):2597-2604. doi: 10.1016/j.jacc.2016.09.966. |
| Apixaban & rivaroxaban | 33226545 | Sun X | Hemorrhage Risk Profiles among Different Antithrombotic Regimens: Evidence from a Real-World Analysis of Postmarketing Surveillance Data | Cardiovasc Drugs Ther. 2022 Feb;36(1):103-112. doi: 10.1007/s10557-020-07110-w. Epub 2020 Nov 23. |
| Apixaban & rivaroxaban | 30383768 | Tepper PG | Real-world comparison of bleeding risks among non-valvular atrial fibrillation patients prescribed apixaban, dabigatran, or rivaroxaban | PLoS One. 2018 Nov 1;13(11):e0205989. doi: 10.1371/journal.pone.0205989. eCollection 2018. |
| Apixaban & rivaroxaban | 30500885 | Villines TC | Comparative safety and effectiveness of dabigatran vs. rivaroxaban and apixaban in patients with non-valvular atrial fibrillation: a retrospective study from a large healthcare system | Eur Heart J Cardiovasc Pharmacother. 2019 Apr 1;5(2):80-90. doi: 10.1093/ehjcvp/pvy044. |
| Apixaban & rivaroxaban | 32640949 | Wetmore JB | Direct-Acting Oral Anticoagulants Versus Warfarin in Medicare Patients With Chronic Kidney Disease and Atrial Fibrillation | Stroke. 2020 Aug;51(8):2364-2373. doi: 10.1161/STROKEAHA.120.028934. Epub 2020 Jul 9. |
| Apixaban & rivaroxaban | 28119380 | Xian Y | Use of Intravenous Recombinant Tissue Plasminogen Activator in Patients With Acute Ischemic Stroke Who Take Non-Vitamin K Antagonist Oral Anticoagulants Before Stroke | Circulation. 2017 Mar 14;135(11):1024-1035. doi: 10.1161/CIRCULATIONAHA.116.023940. Epub 2017 Jan 24. |
| Apixaban & rivaroxaban | 32359718 | Yang L | Real-World Direct Comparison of the Effectiveness and Safety of Apixaban, Dabigatran, Rivaroxaban, and Warfarin in Medicare Beneficiaries With Atrial Fibrillation | Am J Cardiol. 2020 Jul 1;126:29-36. doi: 10.1016/j.amjcard.2020.03.034. Epub 2020 Apr 10. |
| Apixaban & rivaroxaban | 29169468 | Yao X | Renal Outcomes in Anticoagulated Patients With Atrial Fibrillation | J Am Coll Cardiol. 2017 Nov 28;70(21):2621-2632. doi: 10.1016/j.jacc.2017.09.1087. |
| Apixaban & rivaroxaban | 33012172 | Yao X | Comparative Effectiveness and Safety of Oral Anticoagulants Across Kidney Function in Patients With Atrial Fibrillation | Circ Cardiovasc Qual Outcomes. 2020 Oct;13(10):e006515. doi: 10.1161/CIRCOUTCOMES.120.006515. Epub 2020 Oct 5. |
| Apixaban alone | 23883416 | Amin A | Estimated medical cost reductions associated with apixaban in real-world patients with non-valvular atrial fibrillation | J Med Econ. 2013 Oct;16(10):1193-202. doi: 10.3111/13696998.2013.828064. Epub 2013 Aug 7. |
| Apixaban alone | 32989717 | Bradley M | Risk of Stroke and Bleeding in Atrial Fibrillation Treated with Apixaban Compared with Warfarin | J Gen Intern Med. 2020 Dec;35(12):3597-3604. doi: 10.1007/s11606-020-06180-8. Epub 2020 Sep 28. |
| Apixaban alone | 34570310 | Cohen AT | Effectiveness and Safety of Apixaban Versus Warfarin Among Older Patients with Venous Thromboembolism with Different Demographics and Socioeconomic Status | Adv Ther. 2021 Nov;38(11):5519-5533. doi: 10.1007/s12325-021-01918-0. Epub 2021 Sep 27. |
| Apixaban alone | 36149864 | Cohen AT | Effectiveness and safety of apixaban vs warfarin among venous thromboembolism patients at high-risk of bleeding | PLoS One. 2022 Sep 23;17(9):e0274969. doi: 10.1371/journal.pone.0274969. eCollection 2022. |
| Apixaban alone | 29083968 | Deitelzweig S | Effect of Apixaban Versus Warfarin Use on Health Care Resource Utilization and Costs Among Elderly Patients with Nonvalvular Atrial Fibrillation | J Manag Care Spec Pharm. 2017 Nov;23(11):1191-1201. doi: 10.18553/jmcp.2017.17060. Epub 2017 Aug 11. |
| Apixaban alone | 30433823 | Deitelzweig S | Hospital Resource Utilization and Costs Associated With Warfarin Versus Apixaban Treatment Among Patients Hospitalized for Venous Thromboembolism in the United States | Clin Appl Thromb Hemost. 2018 Dec;24(9_suppl):261S-268S. doi: 10.1177/1076029618800806. Epub 2018 Nov 15. |
| Apixaban alone | 34930532 | Deitelzweig S | Effectiveness and Safety of Apixaban Versus Warfarin in Obese Patients with Nonvalvular Atrial Fibrillation Enrolled in Medicare and Veteran Affairs | Am J Cardiol. 2022 Jan 15;163:43-49. doi: 10.1016/j.amjcard.2021.09.047. |
| Apixaban alone | 26213178 | Farr AM | Comparison of hospital length of stay between hospitalized non-valvular atrial fibrillation patients treated with either apixaban or warfarin | Hosp Pract (1995). 2015;43(3):172-9. doi: 10.1080/21548331.2015.1071635. Epub 2015 Jul 27. |
| Apixaban alone | 32452277 | Guo JD | Comparative Clinical and Economic Outcomes Associated with Warfarin Versus Apixaban in the Treatment of Patients with Venous Thromboembolism in a Large U.S. Commercial Claims Database | J Manag Care Spec Pharm. 2020 Aug;26(8):1017-1026. doi: 10.18553/jmcp.2020.19311. Epub 2020 May 26. |
| Apixaban alone | 33348190 | Guo JD | Safety and effectiveness of apixaban compared with warfarin among clinically-relevant subgroups of venous thromboembolism patients in the United States Medicare population | Thromb Res. 2021 Feb;198:163-170. doi: 10.1016/j.thromres.2020.11.039. Epub 2020 Dec 8. |
| Apixaban alone | 31387467 | Hlavacek P | Safety, effectiveness, and health care cost comparisons among elderly patients with venous thromboembolism prescribed warfarin or apixaban in the United States Medicare population | Curr Med Res Opin. 2019 Dec;35(12):2043-2051. doi: 10.1080/03007995.2019.1653067. Epub 2019 Sep 3. |
| Apixaban alone | 35420702 | Kang HR | Effectiveness and Safety of Extended Oral Anticoagulant Therapy in Patients with Venous Thromboembolism: A Retrospective Cohort Study | Clin Pharmacol Ther. 2022 Jul;112(1):133-145. doi: 10.1002/cpt.2611. Epub 2022 May 2. |
| Apixaban alone | 29373602 | Li X | Apixaban 5 and 2.5 mg twice-daily versus warfarin for stroke prevention in nonvalvular atrial fibrillation patients: Comparative effectiveness and safety evaluated using a propensity-score-matched approach | PLoS One. 2018 Jan 26;13(1):e0191722. doi: 10.1371/journal.pone.0191722. eCollection 2018. |
| Apixaban alone | 28300870 | Li XS | Effectiveness and safety of apixaban versus warfarin in non-valvular atrial fibrillation patients in "real-world" clinical practice. A propensity-matched analysis of 76,940 patients | Thromb Haemost. 2017 Jun 2;117(6):1072-1082. doi: 10.1160/TH17-01-0068. Epub 2017 Mar 16. |
| Apixaban alone | 32444398 | Mavrakanas TA | Apixaban versus No Anticoagulation in Patients Undergoing Long-Term Dialysis with Incident Atrial Fibrillation | Clin J Am Soc Nephrol. 2020 Aug 7;15(8):1146-1154. doi: 10.2215/CJN.11650919. Epub 2020 May 22. |
| Apixaban alone | 36200137 | Park H | Comparative effectiveness and safety of extended anticoagulant therapy among Medicare beneficiaries with venous thromboembolism | Clin Transl Sci. 2023 Jan;16(1):128-139. doi: 10.1111/cts.13433. Epub 2022 Oct 17. |
| Apixaban alone | 33421454 | Wetmore JB | CKD Progression in Medicare Beneficiaries With Nonvalvular Atrial Fibrillation Treated With Apixaban Versus Warfarin | Am J Kidney Dis. 2021 Aug;78(2):180-189. doi: 10.1053/j.ajkd.2020.12.004. Epub 2021 Jan 6. |
| Apixaban alone | 35469965 | Wetmore JB | Apixaban Dosing Patterns Versus Warfarin in Patients With Nonvalvular Atrial Fibrillation Receiving Dialysis: A Retrospective Cohort Study | Am J Kidney Dis. 2022 Nov;80(5):569-579.e1. doi: 10.1053/j.ajkd.2022.03.007. Epub 2022 Apr 22. |
| Apixaban alone | 35470214 | Wetmore JB | Apixaban versus Warfarin for Treatment of Venous Thromboembolism in Patients Receiving Long-Term Dialysis | Clin J Am Soc Nephrol. 2022 May;17(5):693-702. doi: 10.2215/CJN.14021021. Epub 2022 Apr 25. |
| Dapagliflozin & sitagliptin | 30101553 | Parker ED | Comparison of healthcare resource utilization and costs in patients with type 2 diabetes initiating dapagliflozin versus sitagliptin | Diabetes Obes Metab. 2019 Feb;21(2):227-233. doi: 10.1111/dom.13502. Epub 2018 Sep 10. |
| Dapagliflozin alone | 28418262 | Cai J | Adherence and persistence in patients with type 2 diabetes mellitus newly initiating canagliflozin, dapagliflozin, dpp-4s, or glp-1s in the United States | Curr Med Res Opin. 2017 Jul;33(7):1317-1328. doi: 10.1080/03007995.2017.1320277. Epub 2017 May 8. |
| Dapagliflozin alone | 36583828 | Danysh HE | Post-Authorization Safety Studies of Acute Liver Injury and Severe Complications of Urinary Tract Infection in Patients with Type 2 Diabetes Exposed to Dapagliflozin in a Real-World Setting | Drug Saf. 2023 Feb;46(2):175-193. doi: 10.1007/s40264-022-01262-4. Epub 2022 Dec 30. |
| Dapagliflozin alone | 36528670 | Johannes CB | Post-Authorization Safety Study of Hospitalization for Acute Kidney Injury in Patients with Type 2 Diabetes Exposed to Dapagliflozin in a Real-World Setting | Drug Saf. 2023 Feb;46(2):157-174. doi: 10.1007/s40264-022-01263-3. Epub 2022 Dec 17. |
| Dapagliflozin alone | 28827404 | Nadkarni GN | Acute Kidney Injury in Patients on SGLT2 Inhibitors: A Propensity-Matched Analysis | Diabetes Care. 2017 Nov;40(11):1479-1485. doi: 10.2337/dc17-1011. Epub 2017 Aug 21. |
| Empagliflozin & dapagliflozin | 28522450 | Kosiborod M | Lower Risk of Heart Failure and Death in Patients Initiated on Sodium-Glucose Cotransporter-2 Inhibitors Versus Other Glucose-Lowering Drugs: The CVD-REAL Study (Comparative Effectiveness of Cardiovascular Outcomes in New Users of Sodium-Glucose Cotransporter-2 Inhibitors) | Circulation. 2017 Jul 18;136(3):249-259. doi: 10.1161/CIRCULATIONAHA.117.029190. Epub 2017 May 18. |
| Empagliflozin & sitagliptin | 36264574 | Htoo PT | Comparative Effectiveness of Empagliflozin vs Liraglutide or Sitagliptin in Older Adults With Diverse Patient Characteristics | JAMA Netw Open. 2022 Oct 3;5(10):e2237606. doi: 10.1001/jamanetworkopen.2022.37606. |
| Empagliflozin & sitagliptin | 30955357 | Patorno E | Empagliflozin and the Risk of Heart Failure Hospitalization in Routine Clinical Care | Circulation. 2019 Jun 18;139(25):2822-2830. doi: 10.1161/CIRCULATIONAHA.118.039177. Epub 2019 Apr 8. |
| Empagliflozin alone | 36705285 | Peasah SK | Real-world impact of empagliflozin on total cost of care in adults with type 2 diabetes: Results from an outcomes-based agreement | J Manag Care Spec Pharm. 2023 Feb;29(2):152-160. doi: 10.18553/jmcp.2023.29.2.152. |
| Etanercept | 23852763 | Baddley JW | Non-viral opportunistic infections in new users of tumour necrosis factor inhibitor therapy: results of the SAfety Assessment of Biologic ThERapy (SABER) study | Ann Rheum Dis. 2014 Nov;73(11):1942-8. doi: 10.1136/annrheumdis-2013-203407. Epub 2013 Jul 13. |
| Etanercept | 26097194 | Bonafede M | Etanercept-Methotrexate Combination Therapy Initiators Have Greater Adherence and Persistence Than Triple Therapy Initiators With Rheumatoid Arthritis | Arthritis Care Res (Hoboken). 2015 Dec;67(12):1656-63. doi: 10.1002/acr.22638. |
| Etanercept | 31997371 | Calip GS | Targets of biologic disease-modifying antirheumatic drugs and risk of multiple myeloma | Int J Cancer. 2020 Sep 1;147(5):1300-1305. doi: 10.1002/ijc.32891. Epub 2020 Feb 8. |
| Etanercept | 24470378 | Curtis JR | Risk of hospitalized bacterial infections associated with biologic treatment among US veterans with rheumatoid arthritis | Arthritis Care Res (Hoboken). 2014 Jul;66(7):990-7. doi: 10.1002/acr.22281. |
| Etanercept | 31108503 | George MD | Risk of Biologics and Glucocorticoids in Patients With Rheumatoid Arthritis Undergoing Arthroplasty: A Cohort Study | Ann Intern Med. 2019 Jun 18;170(12):825-836. doi: 10.7326/M18-2217. Epub 2019 May 21. |
| Etanercept | 17909386 | Grijalva CG | Assessment of adherence to and persistence on disease-modifying antirheumatic drugs (DMARDs) in patients with rheumatoid arthritis | Med Care. 2007 Oct;45(10 Supl 2):S66-76. doi: 10.1097/MLR.0b013e318041384c. |
| Etanercept | 22056398 | Grijalva CG | Initiation of tumor necrosis factor-α antagonists and the risk of hospitalization for infection in patients with autoimmune diseases | JAMA. 2011 Dec 7;306(21):2331-9. doi: 10.1001/jama.2011.1692. Epub 2011 Nov 6. |
| Etanercept | 14577718 | Harley CR | Treatment compliance and dosage administration among rheumatoid arthritis patients receiving infliximab, etanercept, or methotrexate | Am J Manag Care. 2003 Oct;9(6 Suppl):S136-43. |
| Etanercept | 27882833 | Harnett J | Evaluation of Real-World Experience with Tofacitinib Compared with Adalimumab, Etanercept, and Abatacept in RA Patients with 1 Previous Biologic DMARD: Data from a U.S. Administrative Claims Database | J Manag Care Spec Pharm. 2016 Dec;22(12):1457-1471. doi: 10.18553/jmcp.2016.22.12.1457. |
| Etanercept | 29329557 | Harrold LR | One-year risk of serious infection in patients treated with certolizumab pegol as compared with other TNF inhibitors in a real-world setting: data from a national U.S. rheumatoid arthritis registry | Arthritis Res Ther. 2018 Jan 2;20(1):2. doi: 10.1186/s13075-017-1496-5. |
| Etanercept | 24131136 | Joyce AT | National and regional dose escalation and cost of tumor necrosis factor blocker therapy in biologic-naïve rheumatoid arthritis patients in US health plans | J Med Econ. 2014 Jan;17(1):1-10. doi: 10.3111/13696998.2013.856314. Epub 2013 Oct 31. |
| Etanercept | 21358439 | Lane MA | TNF-α antagonist use and risk of hospitalization for infection in a national cohort of veterans with rheumatoid arthritis | Medicine (Baltimore). 2011 Mar;90(2):139-145. doi: 10.1097/MD.0b013e318211106a. |
| Etanercept | 29952710 | Lee S | Evaluating the Effect of Treatment Persistence on the Economic Burden of Moderate to Severe Psoriasis and/or Psoriatic Arthritis Patients in the U.S. Department of Defense Population | J Manag Care Spec Pharm. 2018 Jul;24(7):654-663. doi: 10.18553/jmcp.2018.24.7.654. |
| Etanercept | 34314123 | Maksabedian Hernandez EJ | Association of physician specialty with psoriatic arthritis treatment and costs | Am J Manag Care. 2021 Jul 1;27(7):e226-e233. doi: 10.37765/ajmc.2021.88706. |
| Etanercept | 27159030 | Mamtani R | Association Between Breast Cancer Recurrence and Immunosuppression in Rheumatoid Arthritis and Inflammatory Bowel Disease: A Cohort Study | Arthritis Rheumatol. 2016 Oct;68(10):2403-11. doi: 10.1002/art.39738. |
| Etanercept | 21572150 | Markenson JA | Persistence with anti-tumor necrosis factor therapies in patients with rheumatoid arthritis: observations from the RADIUS registry | J Rheumatol. 2011 Jul;38(7):1273-81. doi: 10.3899/jrheum.101142. Epub 2011 May 15. |
| Etanercept | 35098751 | Murage MJ | Treatment patterns and health care costs among patients with psoriatic arthritis treated with biologic or targeted synthetic disease-modifying antirheumatic drugs | J Manag Care Spec Pharm. 2022 Feb;28(2):206-217. doi: 10.18553/jmcp.2022.28.2.206. |
| Etanercept | 26599370 | Phillips C | Tumor Necrosis Factor Inhibition and Head and Neck Cancer Recurrence and Death in Rheumatoid Arthritis | PLoS One. 2015 Nov 23;10(11):e0143286. doi: 10.1371/journal.pone.0143286. eCollection 2015. |
| Etanercept | 25260564 | Takeshita J | Comparative effectiveness of less commonly used systemic monotherapies and common combination therapies for moderate to severe psoriasis in the clinical setting | J Am Acad Dermatol. 2014 Dec;71(6):1167-75. doi: 10.1016/j.jaad.2014.08.003. Epub 2014 Sep 24. |
| Etanercept | 25201241 | Yun H | Risks of herpes zoster in patients with rheumatoid arthritis according to biologic disease-modifying therapy | Arthritis Care Res (Hoboken). 2015 May;67(5):731-6. doi: 10.1002/acr.22470. |
| Etanercept | 26130274 | Yun H | The comparative effectiveness of biologics among older adults and disabled rheumatoid arthritis patients in the Medicare population | Br J Clin Pharmacol. 2015 Dec;80(6):1447-57. doi: 10.1111/bcp.12709. Epub 2015 Sep 30. |
| Etanercept | 26315675 | Yun H | Comparative Risk of Hospitalized Infection Associated With Biologic Agents in Rheumatoid Arthritis Patients Enrolled in Medicare | Arthritis Rheumatol. 2016 Jan;68(1):56-66. doi: 10.1002/art.39399. |
| Etanercept | 33863840 | Zhang H | Comparative effectiveness of biologics and targeted therapies for psoriatic arthritis | RMD Open. 2021 Apr;7(1):e001399. doi: 10.1136/rmdopen-2020-001399. |
| Etanercept | 25370912 | Zhang J | Impact of biologic agents with and without concomitant methotrexate and at reduced doses in older rheumatoid arthritis patients | Arthritis Care Res (Hoboken). 2015 May;67(5):624-32. doi: 10.1002/acr.22510. |
| Etanercept | 26792814 | Zhang J | Comparative effects of biologics on cardiovascular risk among older patients with rheumatoid arthritis | Ann Rheum Dis. 2016 Oct;75(10):1813-8. doi: 10.1136/annrheumdis-2015-207870. Epub 2016 Jan 20. |
| Etanercept | 32203525 | Zhou M | Tumor Necrosis Factor (TNF) blocking agents are associated with lower risk for Alzheimer's disease in patients with rheumatoid arthritis and psoriasis | PLoS One. 2020 Mar 23;15(3):e0229819. doi: 10.1371/journal.pone.0229819. eCollection 2020. |
| Ibrutinib | 31678080 | Emond B | Comparison of Time to Next Treatment, Health Care Resource Utilization, and Costs in Patients with Chronic Lymphocytic Leukemia Initiated on Front-line Ibrutinib or Chemoimmunotherapy | Clin Lymphoma Myeloma Leuk. 2019 Dec;19(12):763-775.e2. doi: 10.1016/j.clml.2019.08.004. Epub 2019 Aug 26. |
| Ibrutinib | 31208701 | Fradley MG | Rates and Risk of Atrial Arrhythmias in Patients Treated With Ibrutinib Compared With Cytotoxic Chemotherapy | Am J Cardiol. 2019 Aug 15;124(4):539-544. doi: 10.1016/j.amjcard.2019.05.029. Epub 2019 May 25. |
| Ibrutinib | 32880204 | Huang Q | Time to Next Treatment, Health Care Resource Utilization, and Costs Associated with Ibrutinib Use Among U.S. Veterans with Chronic Lymphocytic Leukemia/Small Lymphocytic Lymphoma: A Real-World Retrospective Analysis | J Manag Care Spec Pharm. 2020 Oct;26(10):1266-1275. doi: 10.18553/jmcp.2020.20095. Epub 2020 Sep 3. |
| Ibrutinib | 33044848 | Huang Q | Healthcare resource utilization and costs associated with first-line ibrutinib compared to chemoimmunotherapy treatment among Medicare beneficiaries with chronic lymphocytic leukemia | Curr Med Res Opin. 2020 Dec;36(12):2009-2018. doi: 10.1080/03007995.2020.1835851. Epub 2020 Oct 29. |
| Ibrutinib | 28453705 | Mato AR | Optimal sequencing of ibrutinib, idelalisib, and venetoclax in chronic lymphocytic leukemia: results from a multicenter study of 683 patients | Ann Oncol. 2017 May 1;28(5):1050-1056. doi: 10.1093/annonc/mdx031. |
| Ibrutinib | 26588193 | Thompson PA | β2 -microglobulin normalization within 6 months of ibrutinib-based treatment is associated with superior progression-free survival in patients with chronic lymphocytic leukemia | Cancer. 2016 Feb 15;122(4):565-73. doi: 10.1002/cncr.29794. Epub 2015 Nov 20. |
| Rivaroxaban alone | 25910928 | Abraham NS | Comparative risk of gastrointestinal bleeding with dabigatran, rivaroxaban, and warfarin: population based cohort study | BMJ. 2015 Apr 24;350:h1857. doi: 10.1136/bmj.h1857. |
| Rivaroxaban alone | 30421391 | Alcusky M | Dabigatran Versus Rivaroxaban for Secondary Stroke Prevention in Patients with Atrial Fibrillation Rehabilitated in Skilled Nursing Facilities | Drugs Aging. 2018 Dec;35(12):1089-1098. doi: 10.1007/s40266-018-0610-y. |
| Rivaroxaban alone | 27889397 | Bengtson LGS | Comparative effectiveness of dabigatran and rivaroxaban versus warfarin for the treatment of non-valvular atrial fibrillation | J Cardiol. 2017 Jun;69(6):868-876. doi: 10.1016/j.jjcc.2016.08.010. Epub 2016 Nov 23. |
| Rivaroxaban alone | 36658454 | Berger JS | Healthcare Resource Utilization and Costs of Rivaroxaban Versus Warfarin Among Non-valvular Atrial Fibrillation (NVAF) Patients with Diabetes in a US Population | Adv Ther. 2023 Mar;40(3):1224-1241. doi: 10.1007/s12325-022-02422-9. Epub 2023 Jan 19. |
| Rivaroxaban alone | 29622591 | Briasoulis A | Safety and Efficacy of Novel Oral Anticoagulants Versus Warfarin in Medicare Beneficiaries With Atrial Fibrillation and Valvular Heart Disease | J Am Heart Assoc. 2018 Apr 5;7(8):e008773. doi: 10.1161/JAHA.118.008773. |
| Rivaroxaban alone | 32013886 | Briasoulis A | Characteristics and outcomes in patients with atrial fibrillation receiving direct oral anticoagulants in off-label doses | BMC Cardiovasc Disord. 2020 Feb 3;20(1):42. doi: 10.1186/s12872-020-01340-4. |
| Rivaroxaban alone | 25911526 | Chang HY | Risk of gastrointestinal bleeding associated with oral anticoagulants: population based retrospective cohort study | BMJ. 2015 Apr 24;350:h1585. doi: 10.1136/bmj.h1585. |
| Rivaroxaban alone | 29590141 | Charlton B | Length of hospitalization and mortality for bleeding during treatment with warfarin, dabigatran, or rivaroxaban | PLoS One. 2018 Mar 28;13(3):e0193912. doi: 10.1371/journal.pone.0193912. eCollection 2018. |
| Rivaroxaban alone | 27327275 | Coleman CI | Treatment Persistence and Discontinuation with Rivaroxaban, Dabigatran, and Warfarin for Stroke Prevention in Patients with Non-Valvular Atrial Fibrillation in the United States | PLoS One. 2016 Jun 21;11(6):e0157769. doi: 10.1371/journal.pone.0157769. eCollection 2016. |
| Rivaroxaban alone | 31054829 | Coleman CI | Rivaroxaban Versus Warfarin in Patients With Nonvalvular Atrial Fibrillation and Severe Kidney Disease or Undergoing Hemodialysis | Am J Med. 2019 Sep;132(9):1078-1083. doi: 10.1016/j.amjmed.2019.04.013. Epub 2019 May 2. |
| Rivaroxaban alone | 31549153 | Coleman CI | Effectiveness and safety of rivaroxaban vs. warfarin in patients with non-valvular atrial fibrillation and coronary or peripheral artery disease | Eur Heart J Cardiovasc Pharmacother. 2020 Jul 1;6(3):159-166. doi: 10.1093/ehjcvp/pvz047. |
| Rivaroxaban alone | 30251553 | Gilligan AM | Comparison of stroke- and bleed-related healthcare resource utilization and costs among patients with newly diagnosed non-valvular atrial fibrillation and newly treated with dabigatran, rivaroxaban, or warfarin | Expert Rev Pharmacoecon Outcomes Res. 2019 Apr;19(2):203-212. doi: 10.1080/14737167.2019.1527220. Epub 2018 Oct 4. |
| Rivaroxaban alone | 31005440 | Glassberg MB | Changing Patterns of Anticoagulation After Total Hip Arthroplasty in the United States: Frequency of Deep Vein Thrombosis, Pulmonary Embolism, and Complications With Rivaroxaban and Warfarin | J Arthroplasty. 2019 Aug;34(8):1793-1801. doi: 10.1016/j.arth.2019.03.057. Epub 2019 Mar 31. |
| Rivaroxaban alone | 27695821 | Graham DJ | Stroke, Bleeding, and Mortality Risks in Elderly Medicare Beneficiaries Treated With Dabigatran or Rivaroxaban for Nonvalvular Atrial Fibrillation | JAMA Intern Med. 2016 Nov 1;176(11):1662-1671. doi: 10.1001/jamainternmed.2016.5954. |
| Rivaroxaban alone | 31432074 | Hernandez AV | Rivaroxaban vs. warfarin and renal outcomes in non-valvular atrial fibrillation patients with diabetes | Eur Heart J Qual Care Clin Outcomes. 2020 Oct 1;6(4):301-307. doi: 10.1093/ehjqcco/qcz047. |
| Rivaroxaban alone | 31422520 | Houghton DE | Resolution of acute lower extremity deep vein thrombosis with rivaroxaban compared to warfarin | J Thromb Thrombolysis. 2020 Feb;49(2):199-205. doi: 10.1007/s11239-019-01932-8. |
| Rivaroxaban alone | 33327796 | Korgaonkar S | Comparative effectiveness and safety of non-vitamin-K antagonist oral anticoagulants and warfarin in older adults with atrial fibrillation and diabetes | Curr Med Res Opin. 2021 Mar;37(3):343-356. doi: 10.1080/03007995.2020.1865748. Epub 2021 Jan 13. |
| Rivaroxaban alone | 24758611 | Laliberté F | Is rivaroxaban associated with lower inpatient costs compared to warfarin among patients with non-valvular atrial fibrillation? | Curr Med Res Opin. 2014 Aug;30(8):1521-8. doi: 10.1185/03007995.2014.916159. Epub 2014 May 2. |
| Rivaroxaban alone | 29749269 | Laliberté F | CMS hospital readmission reduction program and anticoagulants received following a total hip and knee arthroplasty discharge | Curr Med Res Opin. 2018 Nov;34(11):1967-1974. doi: 10.1080/03007995.2018.1475347. Epub 2018 Jun 12. |
| Rivaroxaban alone | 25724781 | Lauffenburger JC | Factors driving anticoagulant selection in patients with atrial fibrillation in the United States | Am J Cardiol. 2015 Apr 15;115(8):1095-101. doi: 10.1016/j.amjcard.2015.01.539. Epub 2015 Feb 2. |
| Rivaroxaban alone | 27792638 | Margolis JM | Shorter Hospital Stays and Lower Costs for Rivaroxaban Compared With Warfarin for Venous Thrombosis Admissions | J Am Heart Assoc. 2016 Oct 6;5(10):e003788. doi: 10.1161/JAHA.116.003788. |
| Rivaroxaban alone | 30299591 | Martinez BK | Effectiveness and safety of rivaroxaban vs. warfarin in patients with non-valvular atrial fibrillation and heart failure | ESC Heart Fail. 2019 Feb;6(1):10-15. doi: 10.1002/ehf2.12365. Epub 2018 Oct 9. |
| Rivaroxaban alone | 30597611 | Martinez BK | Influence of Polypharmacy on the Effectiveness and Safety of Rivaroxaban Versus Warfarin in Patients With Nonvalvular Atrial Fibrillation | Pharmacotherapy. 2019 Feb;39(2):196-203. doi: 10.1002/phar.2213. Epub 2019 Jan 28. |
| Rivaroxaban alone | 30646182 | Mentias A | Assessment of Outcomes of Treatment With Oral Anticoagulants in Patients With Atrial Fibrillation and Multiple Chronic Conditions: A Comparative Effectiveness Analysis | JAMA Netw Open. 2018 Sep 7;1(5):e182870. doi: 10.1001/jamanetworkopen.2018.2870. |
| Rivaroxaban alone | 33499689 | Milentijevic D | Healthcare costs before and after stroke in patients with non-valvular atrial fibrillation who initiated treatment with rivaroxaban or warfarin | J Med Econ. 2021 Jan-Dec;24(1):212-217. doi: 10.1080/13696998.2021.1879563. |
| Rivaroxaban alone | 33743312 | Milentijevic D | Risk of Stroke Outcomes in Atrial Fibrillation Patients Treated with Rivaroxaban and Warfarin | J Stroke Cerebrovasc Dis. 2021 May;30(5):105715. doi: 10.1016/j.jstrokecerebrovasdis.2021.105715. Epub 2021 Mar 17. |
| Rivaroxaban alone | 24926732 | Nelson WW | Medication persistence and discontinuation of rivaroxaban versus warfarin among patients with non-valvular atrial fibrillation | Curr Med Res Opin. 2014 Dec;30(12):2461-9. doi: 10.1185/03007995.2014.933577. Epub 2014 Jun 25. |
| Rivaroxaban alone | 28874129 | Norby FL | Comparative effectiveness of rivaroxaban versus warfarin or dabigatran for the treatment of patients with non-valvular atrial fibrillation | BMC Cardiovasc Disord. 2017 Sep 6;17(1):238. doi: 10.1186/s12872-017-0672-5. |
| Rivaroxaban alone | 28408716 | Palamaner Subash Shantha G | Sex-Specific Comparative Effectiveness of Oral Anticoagulants in Elderly Patients With Newly Diagnosed Atrial Fibrillation | Circ Cardiovasc Qual Outcomes. 2017 Apr;10(4):e003418. doi: 10.1161/CIRCOUTCOMES.116.003418. |
| Rivaroxaban alone | 28862952 | Palamaner Subash Shantha G | Sex-Specific Associations of Oral Anticoagulant Use and Cardiovascular Outcomes in Patients With Atrial Fibrillation | J Am Heart Assoc. 2017 Aug 18;6(8):e006381. doi: 10.1161/JAHA.117.006381. |
| Rivaroxaban alone | 29396864 | Streiff MB | Effectiveness and safety of anticoagulants for the treatment of venous thromboembolism in patients with cancer | Am J Hematol. 2018 May;93(5):664-671. doi: 10.1002/ajh.25059. Epub 2018 Feb 23. |
| Rivaroxaban alone | 28712814 | Talukdar A | Safety and efficacy of rivaroxaban compared with warfarin in patients undergoing peripheral arterial procedures | J Vasc Surg. 2017 Oct;66(4):1143-1148. doi: 10.1016/j.jvs.2017.02.052. Epub 2017 Jul 14. |
| Rivaroxaban alone | 29242919 | Uyhazi KE | Association of Novel Oral Antithrombotics With the Risk of Intraocular Bleeding | JAMA Ophthalmol. 2018 Feb 1;136(2):122-130. doi: 10.1001/jamaophthalmol.2017.5677. |
| Rivaroxaban alone | 27548074 | Weeda ER | Rivaroxaban versus Heparin Bridging to Warfarin Therapy: Impact on Hospital Length of Stay and Treatment Costs for Low-Risk Patients with Pulmonary Embolism | Pharmacotherapy. 2016 Oct;36(10):1109-1115. doi: 10.1002/phar.1828. Epub 2016 Sep 19. |
| Sacubitril/valsartan | 31023122 | Albert NM | Lower Hospitalization and Healthcare Costs With Sacubitril/Valsartan Versus Angiotensin-Converting Enzyme Inhibitor or Angiotensin-Receptor Blocker in a Retrospective Analysis of Patients With Heart Failure | J Am Heart Assoc. 2019 May 7;8(9):e011089. doi: 10.1161/JAHA.118.011089. |
| Sacubitril/valsartan | 35525261 | Gilstrap L | Sacubitril/valsartan vs ACEi/ARB at hospital discharge and 5-year survival in older patients with heart failure with reduced ejection fraction: A decision analysis approach | Am Heart J. 2022 Aug;250:23-28. doi: 10.1016/j.ahj.2022.04.007. Epub 2022 May 4. |
| Sacubitril/valsartan | 34350772 | Greene SJ | Clinical Effectiveness of Sacubitril/Valsartan Among Patients Hospitalized for Heart Failure With Reduced Ejection Fraction | J Am Heart Assoc. 2021 Aug 17;10(16):e021459. doi: 10.1161/JAHA.121.021459. Epub 2021 Aug 5. |
| Sacubitril/valsartan | 33901125 | Liang HW | The evolution of guideline-directed medical therapy among decompensated HFrEF patients in sacubitril/valsartan era: Medical expenses and clinical effectiveness | J Chin Med Assoc. 2021 Jun 1;84(6):588-595. doi: 10.1097/JCMA.0000000000000546. |
| Sacubitril/valsartan | 31838035 | Tan NY | Comparative Effectiveness of Sacubitril-Valsartan Versus ACE/ARB Therapy in Heart Failure With Reduced Ejection Fraction | JACC Heart Fail. 2020 Jan;8(1):43-54. doi: 10.1016/j.jchf.2019.08.003. Epub 2019 Dec 11. |
| Sitagliptin alone | 25504156 | Farr AM | Retrospective analysis of long-term adherence to and persistence with DPP-4 inhibitors in US adults with type 2 diabetes mellitus | Adv Ther. 2014 Dec;31(12):1287-305. doi: 10.1007/s12325-014-0171-3. Epub 2014 Dec 12. |
| Sitagliptin alone | 26724938 | Farr AM | Healthcare Costs Among Adults with Type 2 Diabetes Initiating DPP-4 Inhibitors | Adv Ther. 2016 Jan;33(1):68-81. doi: 10.1007/s12325-015-0277-2. Epub 2016 Jan 2. |
| Sitagliptin alone | 37259575 | Fu EL | Sodium-glucose cotransporter 2 inhibitors vs. sitagliptin in heart failure and type 2 diabetes: an observational cohort study | Eur Heart J. 2023 Jun 25;44(24):2216-2230. doi: 10.1093/eurheartj/ehad273. |
| Sitagliptin alone | 19219409 | Lage MJ | Comparison of costs among patients with type 2 diabetes treated with exenatide or sitagliptin therapy | Adv Ther. 2009 Feb;26(2):217-29. doi: 10.1007/s12325-009-0002-0. Epub 2009 Feb 14. |
| Sitagliptin alone | 26938635 | Thayer S | Real-world evaluation of glycemic control among patients with type 2 diabetes mellitus treated with canagliflozin versus dipeptidyl peptidase-4 inhibitors | Curr Med Res Opin. 2016 Jun;32(6):1087-96. doi: 10.1185/03007995.2016.1159954. Epub 2016 Mar 16. |
| Sitagliptin alone | 28943114 | Thayer S | HbA(1c) Outcomes in Patients Treated With Canagliflozin Versus Sitagliptin in US Health Plans | Clin Ther. 2017 Oct;39(10):2061-2072. doi: 10.1016/j.clinthera.2017.08.019. Epub 2017 Sep 22. |
| Sitagliptin alone | 31176543 | Weeda ER | Loop diuretic use among patients with heart failure and type 2 diabetes treated with sodium glucose cotransporter-2 inhibitors | J Diabetes Complications. 2019 Aug;33(8):567-571. doi: 10.1016/j.jdiacomp.2019.05.001. Epub 2019 May 10. |
| Sitagliptin alone | 30554838 | Wysham CH | An investigation into the durability of glycemic control in patients with type II diabetes initiated on canagliflozin or sitagliptin: A real-world analysis of electronic medical records | J Diabetes Complications. 2019 Feb;33(2):140-147. doi: 10.1016/j.jdiacomp.2018.10.016. Epub 2018 Oct 31. |
| Ustekinumab | 35854436 | Cheng D | Comorbidity Influences the Comparative Safety of Biologic Therapy in Older Adults With Inflammatory Bowel Diseases | Am J Gastroenterol. 2022 Nov 1;117(11):1845-1850. doi: 10.14309/ajg.0000000000001907. Epub 2022 Aug 12. |
| Ustekinumab | 33786754 | Torres T | Drug Survival of IL-12/23, IL-17 and IL-23 Inhibitors for Psoriasis Treatment: A Retrospective Multi-Country, Multicentric Cohort Study | Am J Clin Dermatol. 2021 Jul;22(4):567-579. doi: 10.1007/s40257-021-00598-4. Epub 2021 Mar 30. |

**Supplemental Table 3: Indications analyzed among the apixaban and rivaroxaban comparative studies in CMS RWD**

| **Indication** | **Both apixaban & rivaroxaban** | **Apixaban alone** | **Rivaroxaban alone** | **Total** |
| --- | --- | --- | --- | --- |
| AF | 18 (66.7%) | 2 (25.0%) | 2 (40.0%) | 22 (55.0%) |
| AF with comorbidity | 7 (25.9%) | 1 (12.5%) | 2 (40.0%) | 10 (25.0%) |
| VTE | 0 (0.0%) | 5 (62.5%) | 0 (0.0%) | 5 (12.5%) |
| VTE with comorbidity | 1 (3.7%) | 0 (0.0%) | 0 (0.0%) | 1 (2.5%) |
| Indication not specified | 0 (0.0%) | 0 (0.0%) | 1 (20.0%) | 1 (2.5%) |
| Other | 1 (3.7%) | 0 (0.0%) | 0 (0.0%) | 1 (2.5%) |
| Total | 27 (100%) | 8 (100%) | 5 (100%) | 40 (100%) |
| AF = atrial fibrillation, CMS = Centers for Medicare and Medicaid Services, RWD = real-world data, VTE = venous thromboembolism. | | | | |

**Supplemental Table 4: Comparators for the apixaban and rivaroxaban comparative studies in CMS RWD**

|  | **Apixaban** | **Rivaroxaban** | **Warfarin** | **Dabigatran** | **Edoxaban** |
| --- | --- | --- | --- | --- | --- |
| **Apixaban** | - |  |  |  |  |
| **Rivaroxaban** | 27 | - |  |  |  |
| **Warfarin** | 33 | 30 | - |  |  |
| **Dabigatran** | 22 | 25 | N/A | - |  |
| **Edoxaban** | 1 | 1 | N/A | N/A | - |
| Note: studies could include multiple comparators. CMS = Centers for Medicare and Medicaid Services, RWD = real-world data. N/A: warfarin, dabigatran, and edoxaban are not included on the list of drugs that will undergo price negotiations; as a result, comparisons between these drugs were not quantified. | | | | | |
